# Open Globe Injury (OGI) with a Presence of an Intraocular Foreign Body (IOFB)—Epidemiology, Management, and Risk Factors in Long Term Follow-Up

**DOI:** 10.3390/jcm12010190

**Published:** 2022-12-26

**Authors:** Michał Jabłoński, Mateusz Winiarczyk, Katarzyna Biela, Paweł Bieliński, Monika Jasielska, Joanna Batalia, Jerzy Mackiewicz

**Affiliations:** 1 Department of Vitreoretinal Surgery, Medical University of Lublin, Chmielna 1, 20079 Lublin, Poland; 2 Department of Ophthalmology, Provincial Hospital in Zamosc, al. John Paul II 10, 22400 Zamosc, Poland

**Keywords:** intraocular foreign body, IOFB, vitrectomy, ocular trauma, open globe injury, OGI

## Abstract

The purpose of the study was to evaluate visual outcomes and consider management strategies in the eyes with an intraocular foreign body (IOFB). In a single-center, retrospective case-control study, 36 eyes of 36 patients who suffered from open globe injury (OGI) with IOFB were admitted to the Department of Vitreoretinal Surgery of Medical University of Lublin, Poland from January 2015 to December 2020. Most frequent primary procedure was the pars plana vitrectomy (PPV) with IOFB removal (*n* = 28). Retinal detachment (RD) developed in nine eyes soon after injury or as a further complication. Recurrent retinal detachment occurred in eight of these nine cases. Final VA 0.1 or better was observed in 21 eyes (58%). Fifteen patients had BCVA of less than 0.1. One eye was not included in the final VA assessment due to the short follow-up period. In 25 out of 28 patients who underwent any kind of pars plana vitrectomy (ppV) a BCVA of <0.4 was observed. The prognosis after an IOFB injury is uncertain due to multiple factors in a peri- and postoperative period. Factors predisposing to poor visual outcomes are: IOFB localization in the posterior segment, retinal detachment, vitreous hemorrhage and prolonged silicone oil tamponade.

## 1. Introduction

Ocular trauma is a common cause of serious visual impairment, and the open globe injury (OGI) can be additionally complicated by the presence of an intraocular foreign body (IOFB). The most common location of the IOFB is the posterior segment of the eye, which is also correlated with worse prognosis comparing to the anterior segment location. General prognosis in case of the ocular trauma with coexisting IOFB can be classified and described with the Ocular Trauma Score (OTS), and the Birmingham Eye Trauma Terminology (BETT) [1,2].

Mostly affected are young men, hit by a projectile of high velocity. The most common types of injuries connected to the IOFBs are splashing and explosive damage [3,4,5]. The incidence of endophthalmitis, which is associated with a poor visual outcome, tends to be higher in the non-metallic IOFBs [3,6,7].

Although there have been advances in the vitreoretinal techniques and equipment, OGI with IOFB remains a serious issue [8,9]. The purpose of this study was to evaluate the epidemiology, management, and risk factors for poor visual outcomes in patients who suffered such injury.

## 2. Materials and Methods

A retrospective single-center case-series study was conducted on patients admitted to the Department of Vitreoretinal Surgery, Medical University of Lublin, Poland, from January 2015 to December 2020.

A total of 36 patients who suffered from an eye injury with a presence of an IOFB were evaluated. Information regarding each injured eye was collected, including patient information (age, sex), best corrected visual acuity (BCVA) on admission, the initial ocular examination characteristics, the type of IOFB (metallic or non-metallic), localization of the IOFB (anterior or posterior segment of the eye), surgical interventions performed, complications, and BCVA with an intraocular pressure (IOP) during and after treatment. Mean follow-up was 10,4 months (range from 2 to 42 months)

The factors evaluated were the IOFB location, injured structures (cornea, sclera, iris, lens, retina), and postoperative complications (vitreous hemorrhage, choroidal detachment, retinal detachment, development of endophthalmitis, ocular siderosis, or eye atrophy).

## 3. Results

A total of 36 eyes of 36 patients were identified with IOFB injuries. Injuries were found to 15 right and 21 left eyes. Most of the patients were male (*n* = 35) with just one female (*n* = 1). The maximum patient age was 74, and the minimum was 16 years with 37.4 years mean age, and 36.5 years median age.

### 3.1. Clinical Presentation

The wound entry points were: the cornea in 26 eyes, sclera in 6 eyes, and a combined cornea–scleral wound in 4 eyes. The most commonly injured intraocular anatomical structures were the lens (*n =* 26; 72%), the retina (*n =* 20; 56%), and the iris (*n =* 9; 25%). IOFB was localized in the anterior segment (*n =* 9; 25%), posterior segment (*n =* 24; 67%), or combined segments if multiple (*n =* 3; 8%).

The BCVA on admission varied, depending on the severity of the trauma. A total of five patients presented with a BCVA of 0.4 or better. There were 14 patients who presented with a BCVA of >0.1 and <0.4, and 17 patients with worse than 0.1.

### 3.2. Management

All patients underwent surgical globe repair with successful removal of the IOFB. All of the eyes were retained. Initial pars plana vitrectomy (PPV) was performed in 28 eyes (78%). Our standard operating protocol includes 23G vitrectomy, with triamcinolone-assisted complete vitreous removal after assisted indentation of the eyeball. Chandelier illumination is used in selected cases, usually when other surgeons’ assistance is not available. In total, 17 patients underwent combined PPV with phacoemulsification as a primary procedure. A total of seven of them had IOL implantation in the same procedure (41%). Of the overall 20 patients who had IOL implanted at some point of treatment, 6 eyes were left aphakic, and 10 lenses were intact. Most frequent implantation place for the IOL was the ciliary sulcus (*n =* 11). In seven patients, IOL was placed in the posterior lens capsule due to the anatomical capsule residue. Iris Claw type IOL was implanted in three eyes due to the lack of the capsular support. The rest of the patients had another type of globe-repairing procedure. IOFBs were removed in all cases, with only two patients requiring more procedures to remove multiple IOFBs. Most preferred tamponade was silicone oil 5000 in 20 eyes (71%), other applied tamponades included sulfur hexafluoride (SF6) gas (*n =* 4, 14%) and octafluoropropane (C3F8) gas (*n =* 3, 11%).

Additional PPV was required in 18 cases (64%) and 8 patients required more than two procedures. The maximum number of the PPV procedures performed in the same eye amounted to six. The most frequent PPV type was 23G (26 of 28 eyes). Silicone oil removal was performed in 13 out of 20 cases (65%) in the long-term follow-up. Eventually, 11 patients had silicone oil tamponade maintained in the eye due to severe risk of retinal detachment. Decaline was used during any PPV procedure within a group of 23 patients (82%). During the PPV, six patients underwent internal limiting membrane (ILM) peeling. Retinotomy had to be executed in five eyes. In all cases, endolaser was used to secure the retinal breaks. All the procedures used during the vitrectomy are summarized in Table 1.

Retinal detachment (RD) developed in nine eyes soon after injury, or as a further complication. Recurrent retinal detachment occurred in eight out of nine cases. Two patients underwent three episodes of recurrent RD. In nine eyes, vitreous hemorrhage (VH) was observed. Six patients suffered both RD and VH. One patient had RD and choroidal detachment. Other complications such as proliferative vitreoretinopathy (PVR) occurred in seven eyes.

Two patient eyes developed siderosis (the first one at admission and the second patient 4 months after injury) with final BCVA 0.05 and 0.1. In both patients, IOFB was removed immediately after admission, 2 days after the trauma.

**Figure 1:**
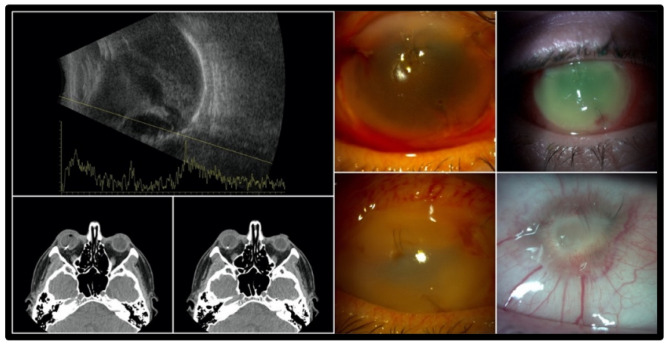
Imaging and clinical follow-up of the IOFB complicated with pan-ophthalmitis leading eventually to the eyeball phthisis (**left side** of the figure). Ultrasound examination (**top left**) showing dense vitreous hemorrhage and choroidal elevation. IOFB is visible as a hyperreflective signal in computer tomography (CT) (**bottom left**).

Endophthalmitis was presented at admission in seven eyes (19%) and no other patient developed endophthalmitis in the future. None of them had RD nor VH. Final VA after endophthalmitis varied from no light perception (*n =* 2) to 0.3. In Figure 1, we present a patient with the worst final outcome of the study group—34 years old male, hit by a metallic projectile from the car wheel. He was admitted three days after injury, with pan-ophthalmitis already present. The CT scan showed a 3 mm metallic IOFB in the posterior segment. Despite the immediate PPV with IOFB removal and silicone oil tamponade, the visual outcome was no light perception (NLP) and eyeball phthisis.

### 3.3. Intraocular Pressure (IOP)

Some patients struggled with elevated IOP (defined as higher than 21 mmHg) during treatment (*n =* 16, 44%) and 31% of them had previous silicone oil tamponade (*n =* 11). However, only two patients had elevated IOP at the last follow-up visit, as it was managed by topical antiglaucoma medications. On the other side, seven eyes had hypotonia (defined as the IOP lower than 6 mmHg).

### 3.4. Surgical Outcomes

Ten injured eyes had a final BCVA of 0.4 or better (28%). Useful vision, defined as >0.1, was achieved in 21 eyes (58%). Fifteen patients had a final BCVA below 0.1, and two of them had no light perception. None of the eyes were enucleated.

Identified factors associated with poor outcome are retinal detachment, proliferative vitreopathy, endophthalmitis, vitreous hemorrhage, and siderosis. Final BCVA of 0.4 or better including one or more of these factors was only experienced by four eyes. Eleven of fifteen patients with at least one risk factor had a final BCVA of less than 0.1. Visual outcomes are summarized in Table 2.

Eyes with maintained silicone oil showed a poor final BCVA. Nine of eleven had a BCVA of less than 0.1.

Final BCVA in eyes with traumatic cataract removal varies. Only four patients who underwent cataract surgery presented BCVA 0.4 or better. The remaining two groups, each eight eyes: first with less than 0.1 and second with more than 0.1 but less than 0.4.

Patients who underwent any PPV displayed a poor final visual outcome, with 14 having a BCVA < 0.1 and 25 of 28 had a BCVA lower than 0.4. On the other hand, six of eight eyes which never had PPV showed a BCVA > 0.4.

## 4. Discussion

The main goal in the management of OGI with IOFB is always to preserve an eye globe and restore ocular integrity. Following targets are to achieve a good visual acuity outcome, and to avoid any further complications. Nowadays, the easier access to the pars plana vitrectomy allowed IOFB management within the posterior segment as a predominant surgical option [10].

Timing of IOFB removal remains a controversy, with inconsistent, often conflicting results [11,12,13,14,15]. As IOFB can present a wide variety of coexisting traumatic wounds and complications, every case is different from the previous one. If possible, we prefer primary wound repair with deferred IOFB removal, usually within 2 to 5 days. This approach gives the best control over a post-traumatic eye, avoiding the open globe vitrectomy, with relatively low risk of endophthalmitis. In this case series, most of the patients had a primary repair completed in other units, had early signs of endophthalmitis, retinal detachment, or were a high suspect of organic IOFB. Therefore, in most of these cases we performed a combined wound repair with simultaneous IOFB removal.

It is important to identify absence of IOFB in OGI. The most reliable method regardless of the localization is a computed tomography (CT) scan. The traditional X-ray of the orbit, and gentle ultrasound examination can also be used as preliminary diagnostic tools, but they will not provide the exact location and size of the IOFB [16].

The existence of the retinal pathology is a significant factor in poor visual acuity. In our study, retinal detachment occurred in nine eyes (25%), and eight of these nine had recurrent retinal detachment. El-Asrar et al. conducted a study of 96 patients in which 6 patients presented with RD at presentation and another 19 after vitrectomy, 25 (26%) in total, which is comparable to our study. Retinal detachment is identified as a factor associated with a poor visual outcome [17].

IOFB presence predispose to the development of the endophthalmitis. In various studies it ranged from 0% to 48.1% [9]. In our study, we observed 19% rate of endophthalmitis (*n =* 7). Endophthalmitis occurred more often in this group than in some previous reports. All the patients received intravitreal antibiotics intraoperatively—either vancomycin in the infusion line during the PPV, or cefuroxime at the end of the procedure if no PPV was executed.

During vitrectomy, intravitreal vancomycin was used as part of the prophylaxis of endophthalmitis. The drug is administered empirically due to the inability to perform a culture and wait for the result before starting targeted treatment. Vancomycin is a glycopeptide antibiotic that acts on gram-positive bacteria, especially penicillin-resistant ones [18]. A study by Borhani et al. found no toxic effects of intravitreal vancomycin on rabbit retinas even at concentrations well above the minimal inhibition concentration [19]. Watanachai et al. administered intravenous vancomycin 1 g every 12 h and ceftazidime 1 g every 8 h for 3 to 5 days and an intravitreal injection of antibiotics at the time of primary repair. Additionally, every patient with endophthalmitis or with high risk of infection had an intravitreal antibiotic injection [20]. However, the incidence of endophthalmitis in our study is similar to that obtained by Watanachai et al. despite differences in prophylaxis (19% vs. 20%).

During our follow-up, seven patients (19%) developed PVR. Cardillo et al. found a lower value of incidence of IOFB and PVR development (11%) with a mean time of onset of 3.1 months [21].

The combined PPV with phacoemulsification as a primary procedure was performed in 61% of all PPVs in our study. Nowadays, the cataract phacoemulsification is a preferred technique of traumatic cataract removal, as it often occurs in young patients with softer lens. In these cases, simple aspiration is sufficient for cataract removal [22]. Sometimes a cataract coexisting with IOFB may be present only as a sectoral lesion, insignificant for visual acuity. In such a scenario, removal of the IOFB might be undertaken sparing the lens [23]. Removing the traumatic cataract is a challenging procedure, as the surgeon can face multiple obstacles, such as the corneal laceration, hyphemia, iris damage, or zonular dialysis. Whether to perform the traumatic lens removal as an early or late procedure should be a choice based on the clinical status and surgeons’ experience [24,25]. Certain issues must be taken into consideration when planning a combined or a two-step procedure. The advantages of combined procedure are lower IOP spikes due to lens swelling, better retina visualization, the elderly patients with coexisting senile cataracts, and the economic aspect. The advantages of separate procedures are: quieter eye and extended time for preparation [26]. Another call to make is whether to implant the IOL promptly. Certainly it helps to avoid the capsular adhesion, on the other hand it is often difficult to select correct IOL power. The calculation of the injured eye is challenging, and quite often it is based on the calculation from the non-injured eye [22]. In another study, it was found that in case of traumatic cataract there was no significant difference in visual outcome between primary and secondary cataract extraction nor IOL implantation [27]. In our center, we usually try to spare the lens in cases where the capsule is intact, and the opacification does not affect the visualization of the posterior segment.

Our finding is that retinal detachment and vitreous hemorrhage are the two most frequent complications of injuries complicated with the IOFB presence (both *n =* 9). It is consistent with other reports, and supports the theory that the late complications of OGI can be at least as dire as the original injury [7,8,9,17].

## 5. Conclusions

IOFB remains one of a few ophthalmic true emergencies, needing immediate surgical treatment. Nevertheless, even with the appropriate and well-timed approach, IOFB complications can lead to severe visual impairment, and blindness. Major factors predisposing for worse visual outcome are localization in the posterior segment, retinal detachment, vitreous hemorrhage, and prolonged silicone oil tamponade. Localization of the IOFB in the anterior segment is correlated with better visual prognosis. Proper education and awareness of the predisposed subjects is crucial for avoiding ocular injuries.

## Figures and Tables

**Table 1:** Summary of all the surgical procedures during vitrectomy.

Surgical Procedure	Count	%
PPV	28	100
−23 G	26	93
−25 G Silicone oil Silicone oil removal SF6 C3F8 Decaline ILM peeling PPV + phacoemulsification as 1st procedure	5 20 13 4 3 23 6 17	18 71 46 14 11 82 21 61
IOL implantation in 1st procedure	7	25
Retinotomy	5	18

ILM—Internal Limiting Membrane; PPV—Pars Plana Vitrectomy; IOL—Intraocular Lens.

**Table 2:** Visual outcomes at the last follow-up.

VA Final (All Cases)		VA Final if RF * Present	
≥0.4	10	≥0.4	4
≥0.1	21	≥0.1	7
<0.1	15	<0.1	11

VA—Visual Acuity. * With 1 or more risk factors (RF) present: retinal detachment, Proliferative Vitreoretinopathy (PVR), endophthalmitis, siderosis, vitreous hemorrhage.

## Data Availability

Not applicable.

## References

[1] Kuhn F., Maisiak R., Mann L., Mester V., Morris R., Witherspoon C. (2002). The Ocular Trauma Score (OTS). Ophthalmol. Clin. North Am..

[2] Kuhn F., Morris R., Witherspoon C., Mester V. (2004). The Birmingham Eye Trauma Terminology system (BETT). J. Fr. Ophtalmol..

[3] Duan F., Yuan Z., Liao J., Zheng Y., Yang Y., Lin X. Incidence and Risk Factors of Intraocular Foreign Body-Related Endophthalmitis in Southern China. https://www.hindawi.com/journals/joph/2018/8959108/.

[4] Li L., Lu H., Ma K., Li Y.-Y., Wang H.-Y., Liu N.-P. Etiologic Causes and Epidemiological Characteristics of Patients with Intraocular Foreign Bodies: Retrospective Analysis of 1340 Cases over Ten Years. https://www.hindawi.com/journals/joph/2018/6309638/.

[5] Liu C.C.H., Tong J.M.K., Li P.S.H., Li K.K.W. (2016). Epidemiology and clinical outcome of intraocular foreign bodies in Hong Kong: A 13-year review. Int. Ophthalmol..

[6] Essex R., Yi Q., Charles P.G., Allen P.J. (2004). Post-traumatic endophthalmitis. Ophthalmology.

[7] Jonas J.B., Knorr H.L., Budde W.M. (2000). Prognostic factors in ocular injuries caused by intraocular or retrobulbar foreign bodies. Ophthalmology.

[8] Nicoară S.D., Irimescu I., Calinici T., Cristian C. (2015). Intraocular foreign bodies extracted by pars plana vitrectomy: Clinical characteristics, management, outcomes and prognostic factors. BMC Ophthalmol..

[9] Loporchio D., Mukkamala L., Gorukanti K., Zarbin M., Langer P., Bhagat N. (2016). Intraocular foreign bodies: A review. Surv. Ophthalmol..

[10] Brinton G.S., Aaberg T.M., Reeser F.H., Topping T.M., Abrams G.W. (1982). Surgical Results in Ocular Trauma Involving the Posterior Segment. Am. J. Ophthalmol..

[11] Liu Y., Wang S., Li Y., Gong Q., Su G., Zhao J. (2019). Intraocular Foreign Bodies: Clinical Characteristics and Prognostic Factors Influencing Visual Outcome and Globe Survival in 373 Eyes. J. Ophthalmol..

[12] Keil J.M., Zhao P.Y., Durrani A.F., Azzouz L., Huvard M.J., Dedania V.S., Zacks D.N. (2022). Endophthalmitis, Visual Outcomes, and Management Strategies in Eyes with Intraocular Foreign Bodies. Clin. Ophthalmol..

[13] Colyer M.H., Weber E.D., Weichel E.D., Dick J.S., Bower K.S., Ward T.P., Haller J.A. (2007). Delayed Intraocular Foreign Body Removal without Endophthalmitis during Operations Iraqi Freedom and Enduring Freedom. Ophthalmology.

[14] Parke D.W., Flynn H.W., Fisher Y.L. (2013). Management of intraocular foreign bodies: A clinical flight plan. Can. J. Ophthalmol..

[15] Anguita R., Moya R., Saez V., Bhardwaj G., Salinas A., Kobus R., Nazar C., Manriquez R., Charteris D.G. (2020). Clinical presentations and surgical outcomes of intraocular foreign body presenting to an ocular trauma unit. Graefe’s Arch. Clin. Exp. Ophthalmol..

[16] Patel S., Langer P.D., Zarbin M.A., Bhagat N. (2012). Diagnostic Value of Clinical Examination and Radiographic Imaging in Identification of Intraocular Foreign Bodies in Open Globe Injury. Eur. J. Ophthalmol..

[17] Abu El-Asrar A.M., Al-Amro S.A., Khan N.M., Kangave D. (1998). Retinal detachment after posterior segment intraocular foreign body injuries. Int. Ophthalmol..

[18] Penha F.M., Rodrigues E.B., Furlani B.A., Dib E., Melo G.B., Farah M.E. (2011). Toxicological considerations for intravitreal drugs. Expert Opin. Drug Metab. Toxicol..

[19] Borhani H., Peyman G.A., Wafapoor H. (1993). Use of vancomycin in vitrectomy infusion solution and evaluation of retinal toxicity. Int. Ophthalmol..

[20] Watanachai N., Choovuthayakorn J., Chokesuwattanaskul S., Photcharapongsakul C., Wongsirimeteekul P., Phinyo P., Chaikitmongkol V., Kunavisarut P., Supreeyathitikul P., Patikulsila D. (2021). Risk factors and outcomes of post-traumatic endophthalmitis: A retrospective single-center study. J. Ophthalmic Inflamm. Infect..

[21] Cardillo J.A., Stout J.T., LaBree L., Azen S.P., Omphroy L., Cui J.Z., Kimura H., Hinton D.R., Ryan S.J. (1997). Post-traumatic Proliferative Vitreoretinopathy. Ophthalmology.

[22] Lam D.S.C., Tham C.C.Y., Kwok A.K.H., Gopal L. (1998). Combined phacoemulsification, pars plana vitrectomy, removal of intraocular foreign body (IOFB), and primary intraocular lens implantation for patients with IOFB and traumatic cataract. Eye.

[23] Pieramici D.J., Capone A., Rubsamen P.E., Roseman R.L. (1996). Lens Preservation after Intraocular Foreign Body Injuries. Ophthalmology.

[24] Agarwal A., Kumar D.A., Nair V. (2010). Cataract surgery in the setting of trauma. Curr. Opin. Ophthalmol..

[25] Tabatabaei S.A., Rajabi M.B., Soleimani M., Rahimi F., Yaseri M. (2017). Early versus late traumatic cataract surgery and intraocular lens implantation. Eye.

[26] Kuhn F. (2010). Traumatic cataract: What, when, how. Graefe’s Arch. Clin. Exp. Ophthalmol..

[27] Rumelt S., Rehany U. (2010). The influence of surgery and intraocular lens implantation timing on visual outcome in traumatic cataract. Graefe’s Arch. Clin. Exp. Ophthalmol..

